# Superior frontal sulcus: a non-eloquent corridor for cavernomas of the internal capsule

**DOI:** 10.3389/fneur.2024.1355338

**Published:** 2024-05-01

**Authors:** Felipe Salvagni Pereira, Luis Ángel Canache Jiménez, Edgar David Tenelema Aguaisa, Rene Alejandro Apaza-Tintaya, Luis Gustavo Biondi-Soares, Alexander Feliciano Vilcahuamán Paitán, Pedro Henrique Teixeira Soto, Raphael Wuo-Silva, Feres Chaddad-Neto

**Affiliations:** ^1^Department of Neurology and Neurosurgery, Universidade Federal de São Paulo, São Paulo, Brazil; ^2^Department of Neurosurgery, Hospital Beneficência Portuguesa de São Paulo, São Paulo, Brazil

**Keywords:** cavernous angioma, cavernoma, internal capsule, frontal craniotomy, superior frontal sulcus

## Abstract

**Introduction:**

Deep cavernomas of eloquent areas, located in the region of the basal nuclei and thalamus, account for 9 to 36% of these encephalic vascular malformations. Internal capsule cavernomas are particularly challenging, as they are surrounded by important projection fibers and their manipulation can lead to permanent deficits. To demonstrate through surgical cases that cavernomas of the internal capsule can be approached by frontal craniotomy, via the superior frontal sulcus, in a curative manner and with low morbidity.

**Methods:**

We presented two cases of cavernomas of the internal capsule operated, whose treatment was microsurgical resection via frontal craniotomy and access to the lesion via the superior frontal sulcus, described step-by-step. To elucidate the rationale behind the decision, we used preoperative images with an emphasis on the patients’ tractography and the importance of comparing these images with anatomical specimens dissected in the neuroanatomy and microsurgery laboratory.

**Results:**

The two cases of internal capsule cavernomas, one in the anterior limb and the other in the posterior limb, were treated surgically via the superior frontal sulcus.

**Discussion:**

Both patients showed radiological cure and clinical improvement in the post-operative segment. The patient consented to the procedure and to the publication of his/her image. Treatment of internal capsule cavernomas via the superior frontal sulcus has proven to be a safe and effective option.

## 1 Introduction

Cavernous angioma or cavernoma is a sinusoidal dilatation covered by a single layer of endothelium, separated by a collagen matrix with elastin and smooth muscle (1), which gives it the appearance of small, agglomerated caverns when observed macroscopically. Unlike other vascular pathologies, such as aneurysms, cavernomas are lesions with low blood flow, so angiography hides them.

The prevalence in the general population is estimated to be around 0.4 to 0.9% (2), representing around 5 to 10% of all vascular malformations (3). They can be asymptomatic, cause epilepsy, cerebral hemorrhage, or even develop a new neurological deficit without hemorrhage (4–8). A 2016 meta-analysis was carried out to predict the natural history of untreated cerebral cavernomas. Of 1,620 patients, 204 presented cerebral hemorrhage, with an estimated risk of 15.8% over 5 years (9).

Some studies indicate a 9–35% incidence of deep cavernomas in the brainstem, thalamus, and basal ganglia (10–12). A case series of 176 patients reported that the most common symptoms of these deep lesions are cranial nerve deficit (51.1%), hemiparesis (40.9%), paresthesia (34.7%), and cerebellar symptoms (38.6%), and concluded that these lesions, after the first episode of bleeding, have very aggressive behavior, with high rates of rebleeding. However, they present excellent results of surgical resection and modified Rankin in the long term (13). Another study demonstrated that over 227 patients with basal ganglia and thalamic cavernomas showed improvement with surgical treatment, with a high cure rate and a low of postoperative complications (14).

Similarly, internal capsule cavernomas are particularly challenging, as they have a deep topography, surrounded by eloquent fibers, requiring complex reasoning when deciding on surgical approaches (15–17).

This paper reports a series of two cases of internal capsule cavernomas, one located in the anterior limb and the other in the posterior limb, treated surgically by frontal craniotomy and total resection via the superior frontal sulcus, which we will explain step by step for its implementation, as well as the rationale behind the decision.

## 2 Frontal approach via the superior frontal sulcus

### 2.1 Positioning, craniotomy, and brain exposure

**Figure 1:**
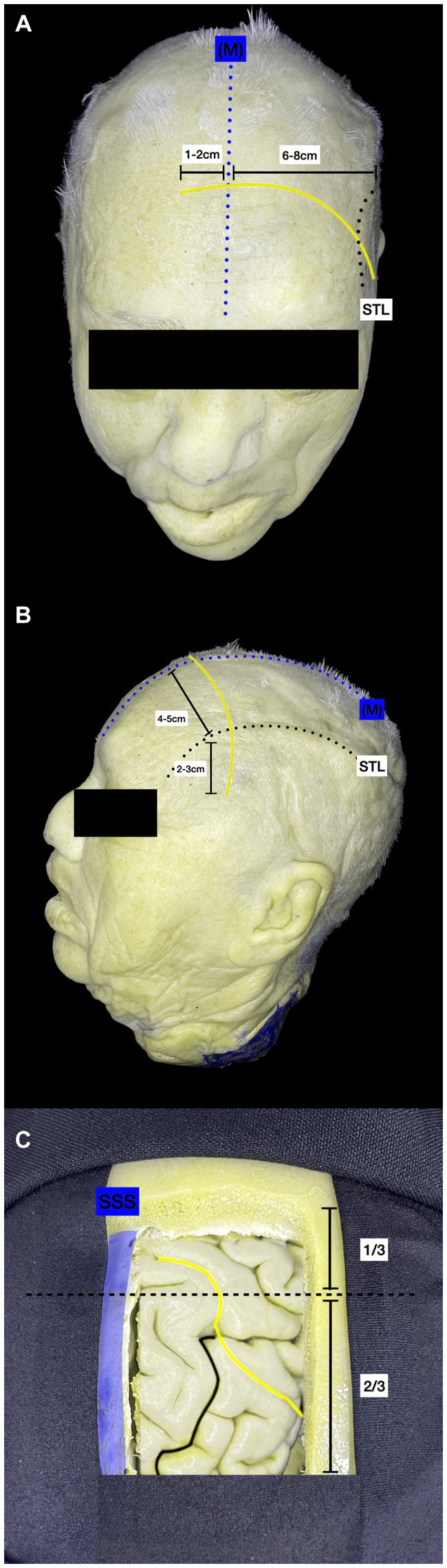
Left frontal craniotomy. **(A)** Anterosuperior view: curvilinear incision (red) crossing both the midline as the medial limit (blue dotted line) and the STL (black dotted line) as the lateral limit. **(B)** Lateral view of the skin incision planning. **(C)** Craniotomy performed: exposure of the SSS (blue), superior frontal sulcus (green) and pre-central sulcus (red) M, midline; STL, superior temporal line; SSS, superior sagittal sinus; cm, centimeters.

We positioned the patient in dorsal decubitus with the head elevated 10 degrees in a neutral position. A curve 6 to 8 cm skin incision was performed over the coronary suture ipsilateral to the lesion, crossing the midline by 1 to 2 cm and the superior temporal line by 2 to 3 cm (Figures 1A,B) to avoid excessive traction on the skin.

The frontal craniotomy should expose the superior frontal gyrus (SFG), middle frontal gyrus (MFG), precentral frontal gyrus (PCG), superior frontal sulcus (SFS), and precentral frontal sulcus (PCS), and interhemispheric fissure (Figure 1C).

The goal is to recognize the structures mentioned above to begin the intersulcal dissection at the superior frontal sulcus and the precentral sulcus intersection.

The procedure is performed under general anesthesia and intraoperative neuro-monitoring by a neurophysiologist.

## 3 Illustrative cases

### 3.1 Case 1: anterior limb of internal capsule cavernoma

A 26-year-old female with no medical history experienced a sudden onset headache. She had a Glasgow Coma Scale (GCS) score of 15 points with MRC grade 3 on the left side.

**Figure 2:**
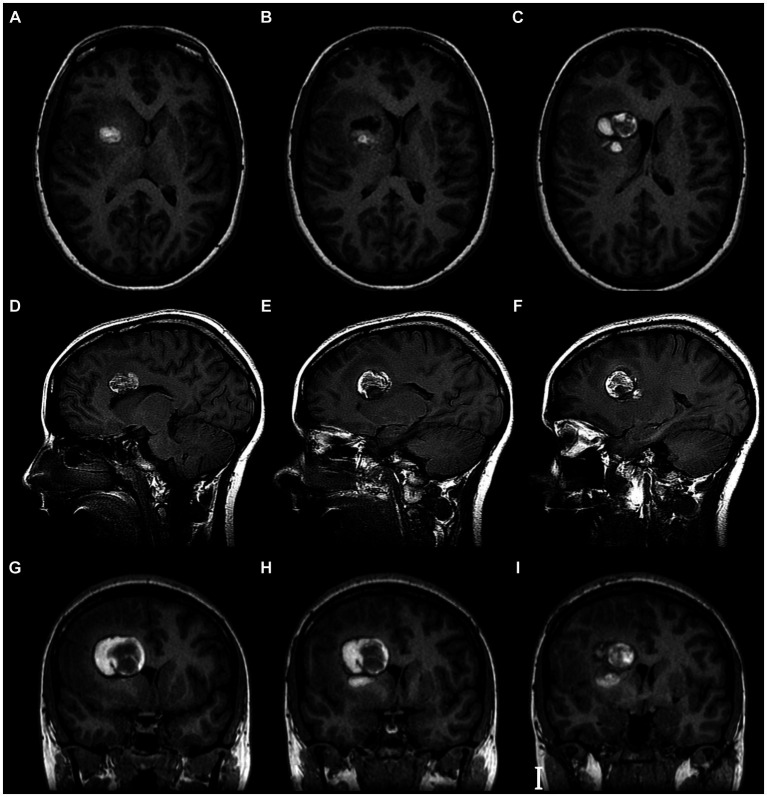
Brain MRI. T1 with Contrast of cavernoma of the anterior limb of the internal capsule. **(A–C)** Axial view. **(D–F)** Sagittal view. **(G–I)** Coronal view. Evidence of hypertensity image, heterogeneous, oval, with circumferential peripheral enhancement. It is intimately related to the anterior limb of the internal capsule, the head of the caudate nucleus, and the anterior horn of the right lateral ventricle.

**Figure 3:**
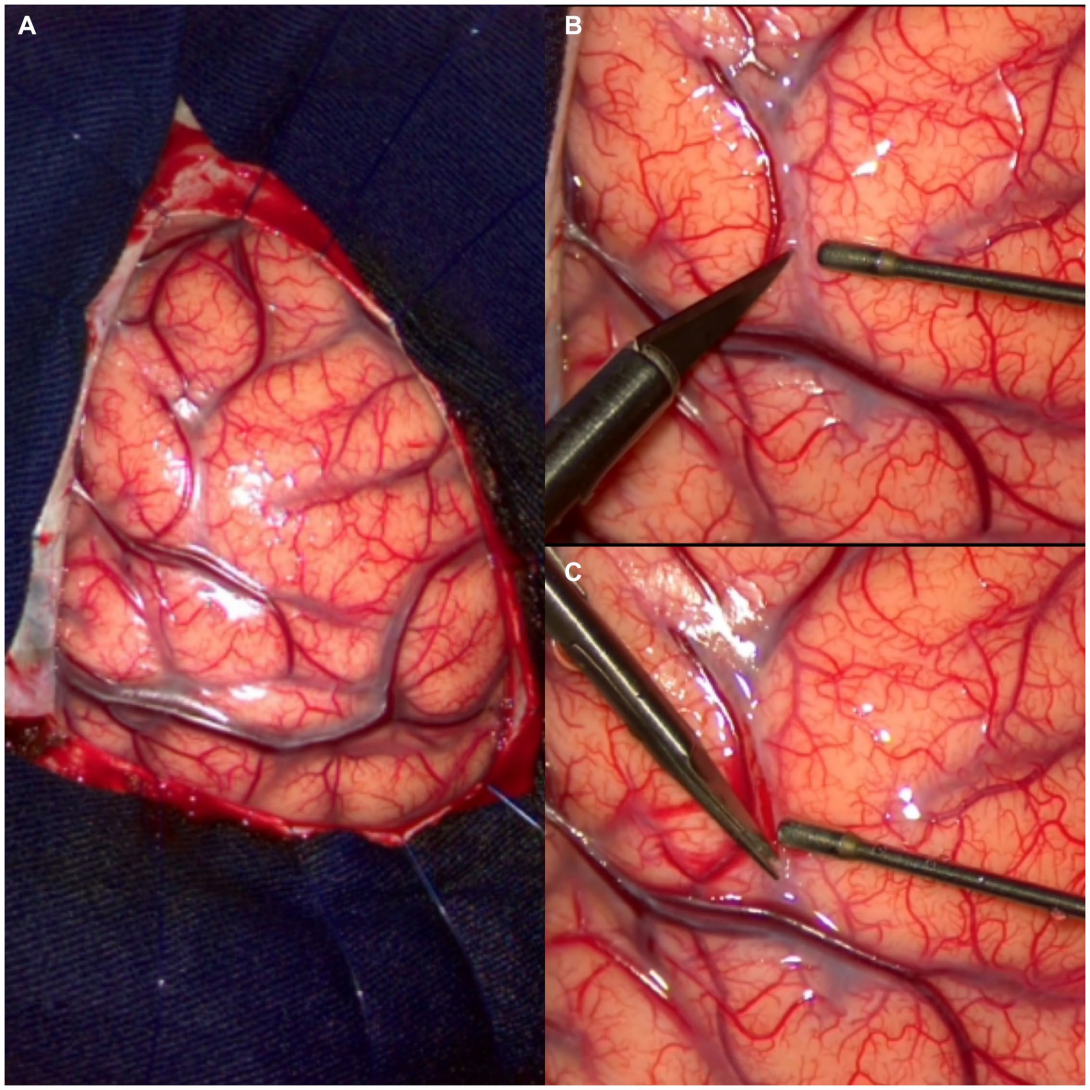
Intraoperative vision. **(A)** Cerebral surgical area exposed at the level of the superior surface of the frontal lobe. **(B,C)** Initial intraductal arachnoid dissection.

**Figure 4:**
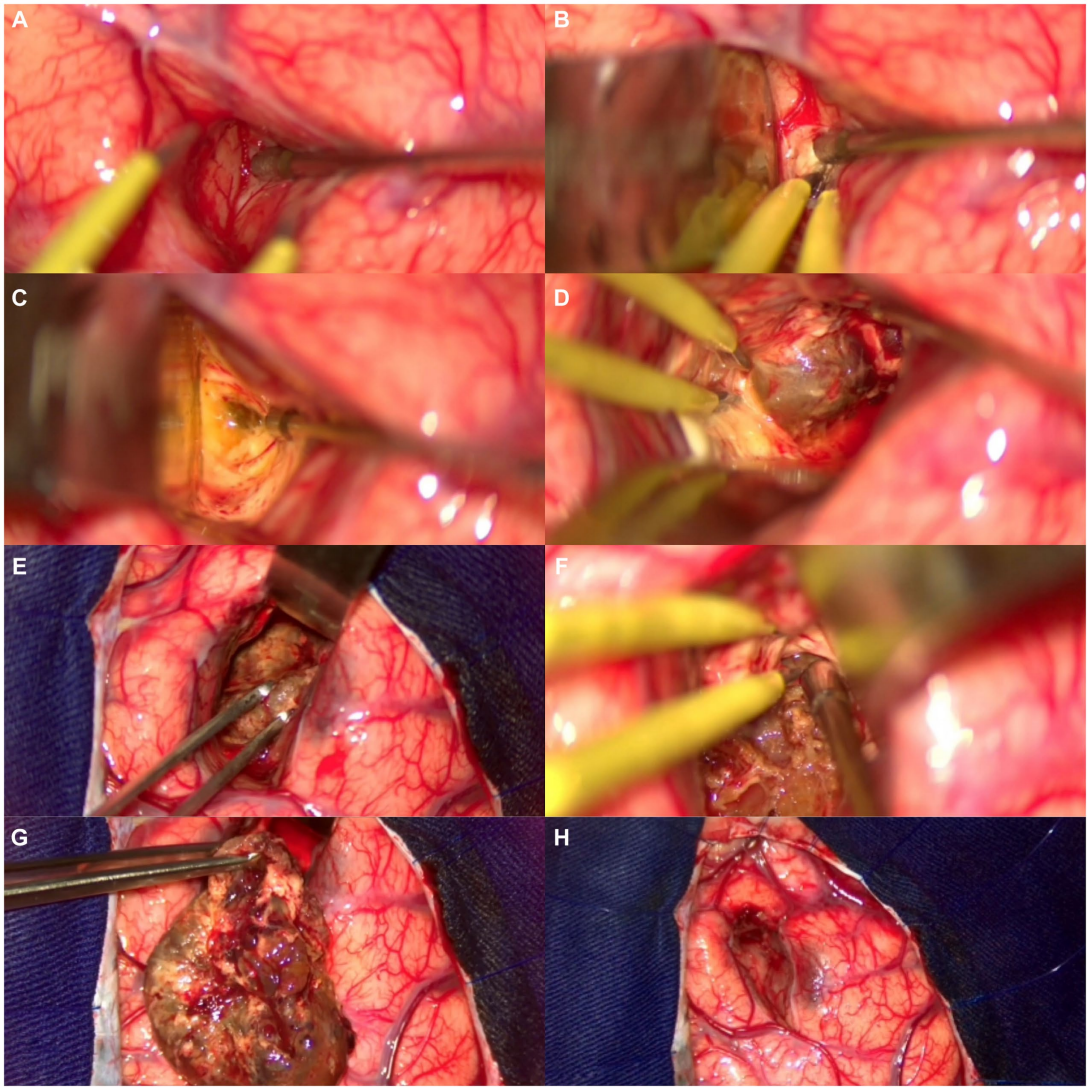
Step-by-step resection of the cavernoma. **(A)** Intersulcal dissection—superior frontal sulcus. **(B)** White substance reflecting the light of the microscope. **(C)** Yellowish hemosiderin deposit, suggestive of recent hemorrhage. **(D)** Circumferential dissection of the cavernoma. **(E)** Careful traction of the cavernoma for 360-degree assessment. **(F)** Disconnection of fibrosis from the lower portion of the cavernoma. **(G)** En bloc resection of the lesion. **(H)** Final appearance after resection of the lesion, cortex, and venous drainage of the region preserved.

An initial neuroimaging study showed parenchymal hemorrhage at the light basal ganglia. MRI showed a lesion in the anterior limb of the internal capsule (Figure 2), and a microsurgical treatment was proposed. The images in Figure 3 show the brain’s exposure after the right frontal craniotomy. We performed an en bloc resection of the cavernoma through the superior frontal sulcus to preserve the adjacent cortical, subcortical, and vascular structures (Figure 4).

**Figure 5:**
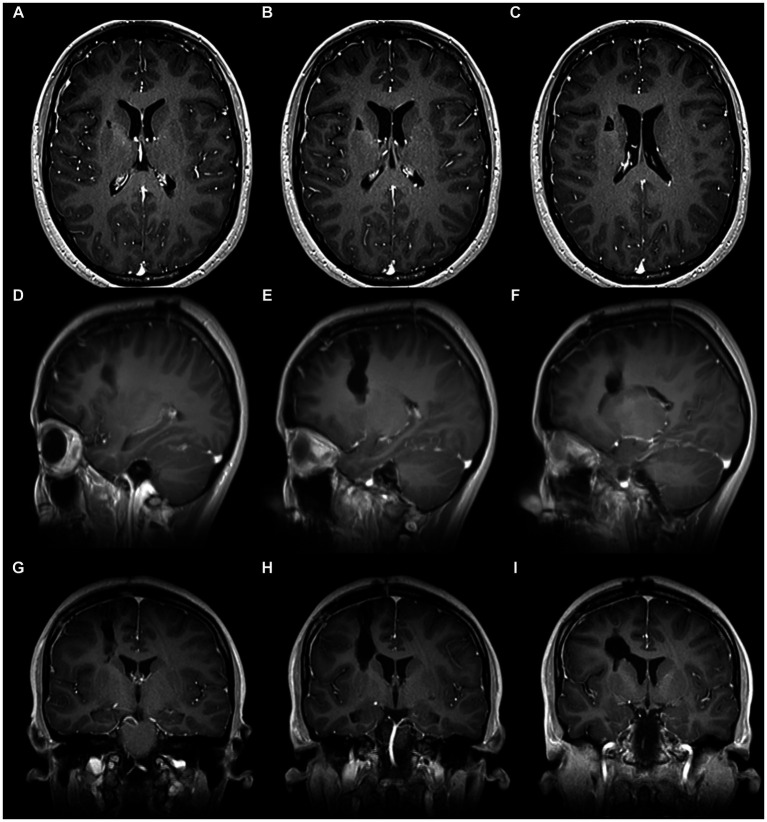
Postoperative brain MRI of cavernoma of the anterior limb of the internal capsule. **(A–C)** Axial view. **(D–F)** Sagittal view. **(G–I)** Coronal view. It shows the resection of the lesion in the depth of the central core and anterior limb of the internal capsule. Note the trajectory from the cerebral surface to the depth, which indicates a dissection through a safe anatomical plane with minimal involvement of noble structures.

The patient evolved with partial symptom improvement in the immediate postoperative period. At 6 months follow-up, he showed complete recovery of symptoms, scoring 0 on the modified Rankin scale (mRS). The postoperative MRI showed complete resection of the lesion (Figure 5).

### 3.2 Case 2: posterior limb of internal capsule cavernoma

A 52-year-old Caucasian female, previously diagnosed with left basal ganglia cavernous malformation, presented with a chronic headache. The symptoms suddenly progressed to right hemiparesis and dysphasia, MRC grade 2 power on the right side.

**Figure 6:**
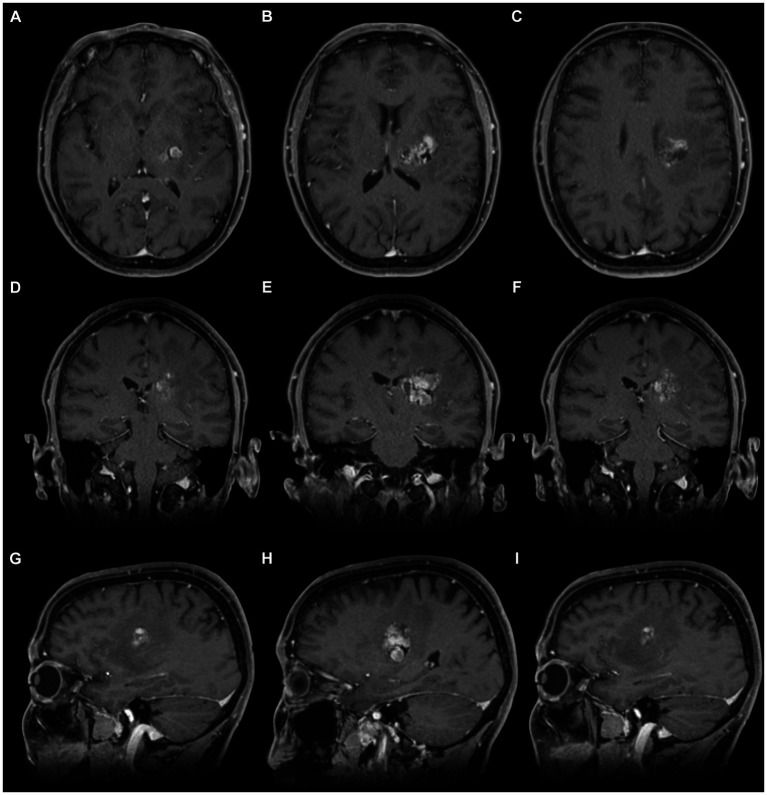
Brain magnetic resonance imaging (T1). **(A–C)** Axial section. **(D–F)** Coronal section. **(G–I)** Sagittal section, where the cavernoma (hypertensity image—heterogeneous) is observed at the level of the posterior limb of the internal capsule. Note its superficial lateral relationship with the posterior aspect of the insula in the sagittal section. The location is posterior to the knee of the internal capsule and the foramen of Monro in the axial section. The arrangement is parallel and inferior to the body of the lateral ventricle in the coronal section.

The MRI showed a left basal ganglia cavernoma in the poster limb of the internal capsule (Figure 6), and the same microsurgical treatment was proposed.

**Figure 7:**
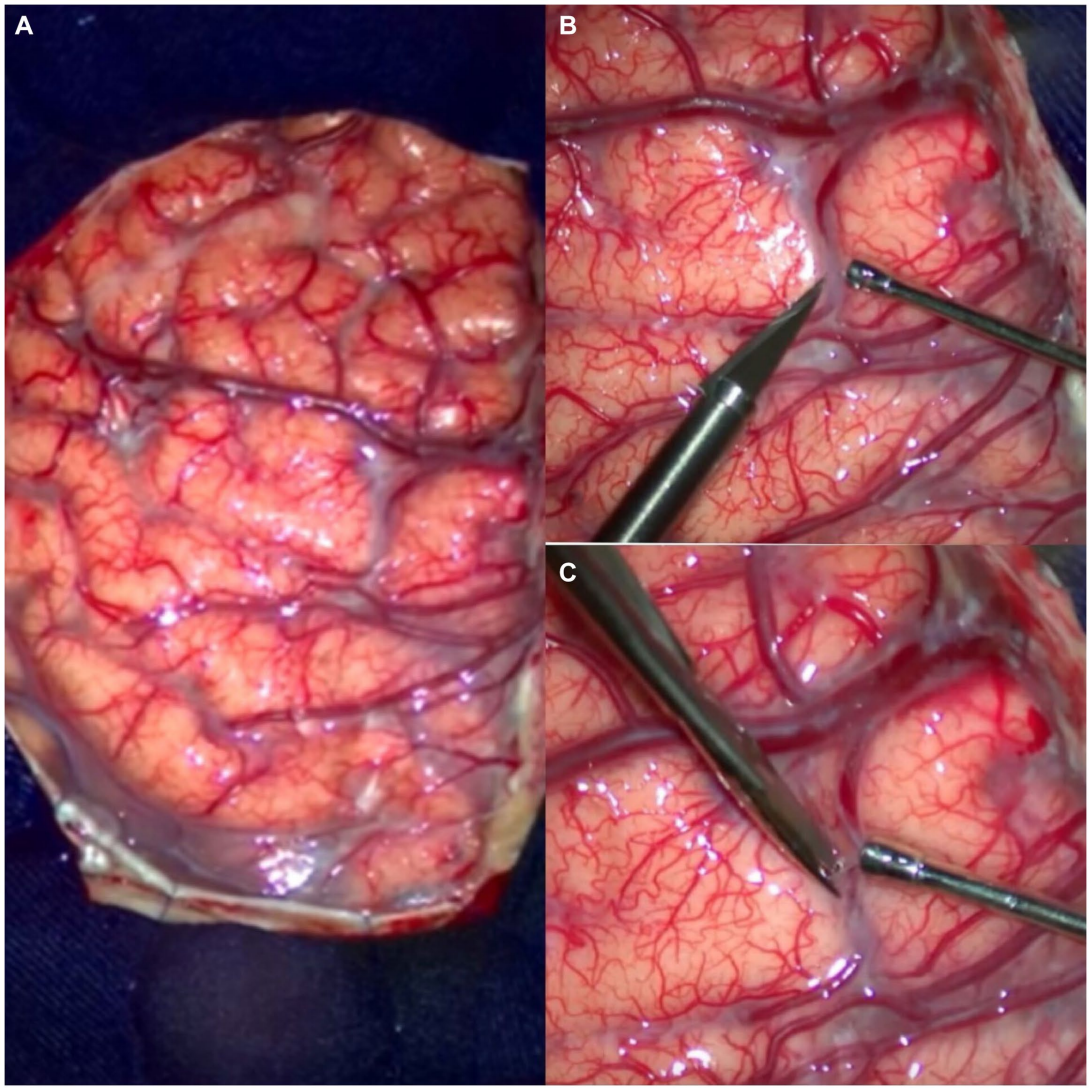
Initial surgical view in the dissection of the case 2 of cavernoma of the posterior limb of the internal capsule. **(A)** Craniotomy with exposure of the frontal and parietal lobes. **(B,C)** Initial arachnoid dissection with “knife and microscissor” over the superior frontal sulcus, close to the precentral sulcus. Note the opening between the superior and middle frontal gyrus.

**Figure 8:**
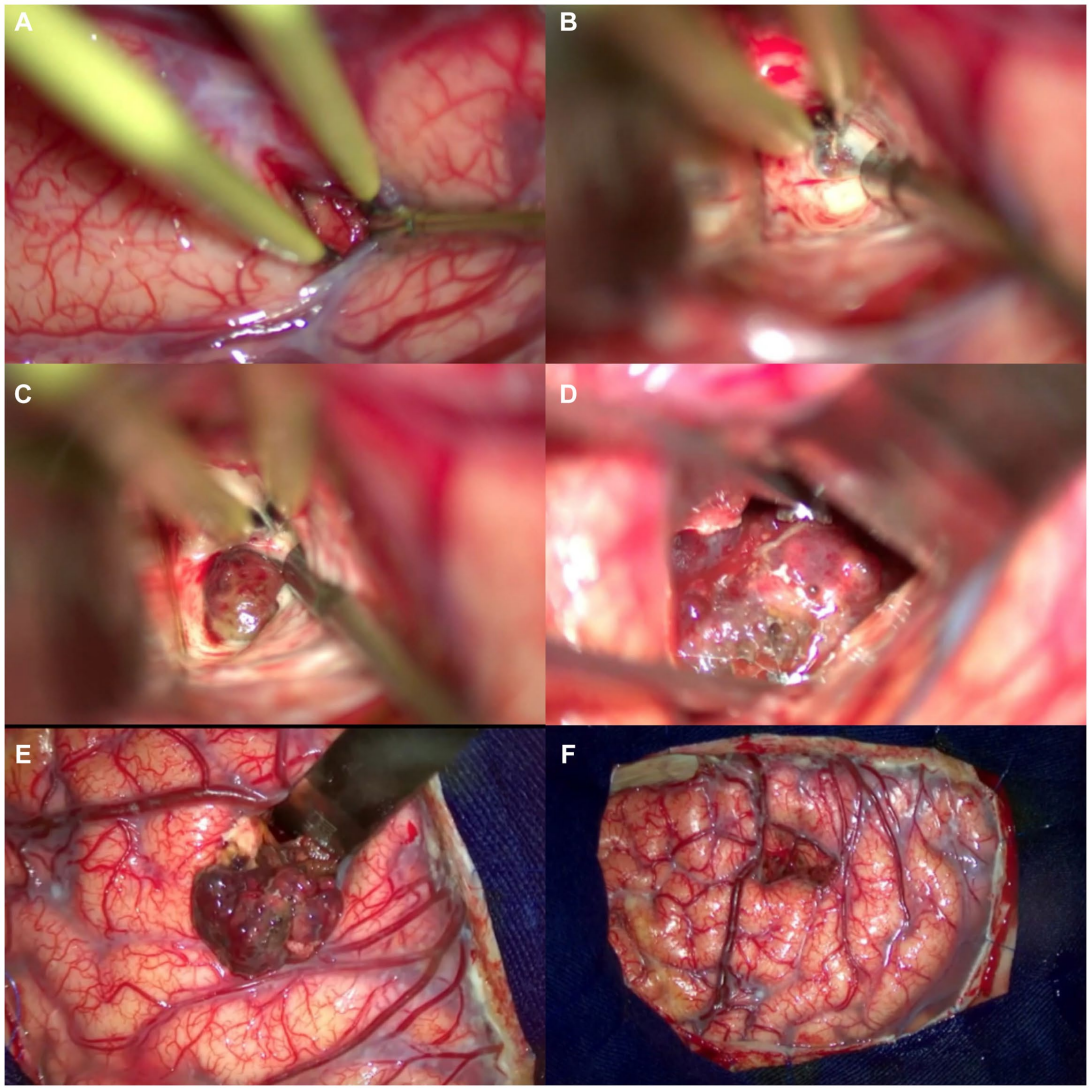
Detailed description of the microsurgical resection of the cavernoma of the posterior limb of the internal capsule. **(A)** Intrasulcular dissection. **(B)** White substance with “high reflection to the microscope’s light.” **(C)** Circumferential dissection of the cavernoma. **(D)** En bloc removal. **(E)** Final aspect of the lesion after its resection. **(F)** The final aspect is the preserved cortex and venous drainage of the region. Note the minimal invasion and aggression to the brain after surgery.

The Figure 7 shows the exposure of the brain after a left frontal craniotomy; in Figure 8, we can see an “en bloc” resection of the cavernoma and the final aspect of the brain.

**Figure 9:**
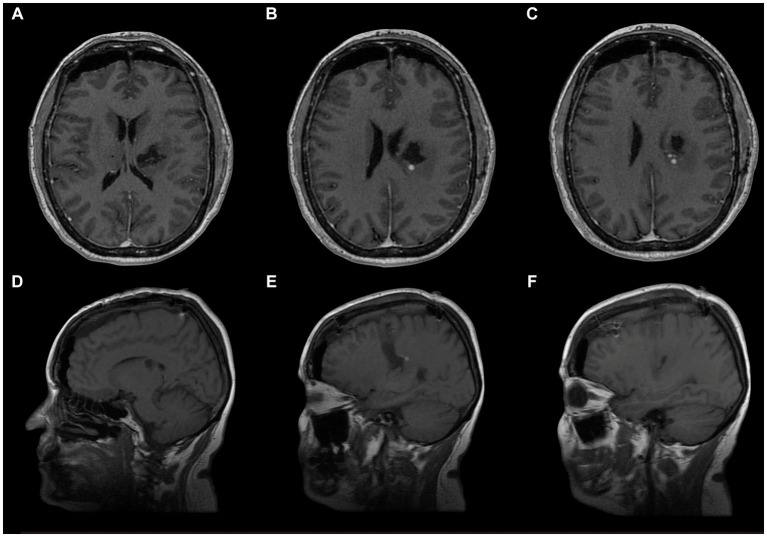
Postoperative MRI of the surgical resection of the cavernoma of the posterior limb of the internal capsule. **(A–C)** Axial view. **(D–F)** Sagittal view. Note the hypointense image suggesting complete resection of the cavernoma and structural indemnity of the adjacent anatomy.

The patient showed worsening symptoms in the postoperative examination, progressing to right hemiplegia and aphasia. At 6 months follow-up, the patient recovered from aphasia and could walk supported by a cane, scoring 1 on the modified Rankin scale (mRS). The postoperative MRI showed complete resection of the lesion (Figure 9).

## 4 Discussion

### 4.1 Cortical structures

**Figure 10:**
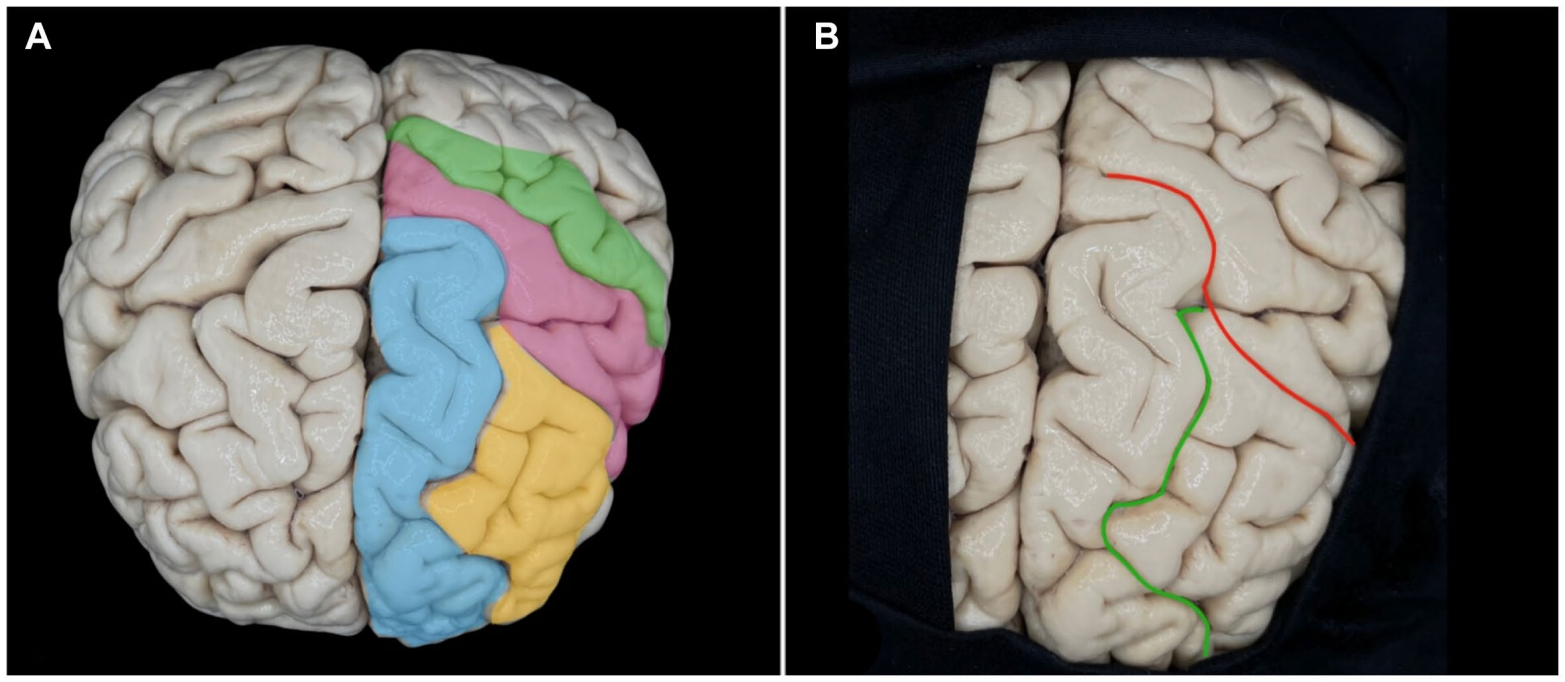
Anatomy of the frontal lobe. **(A)** Schematization of the superior (blue) and middle (yellow) frontal gyri about the precentral (pink) and postcentral (green) gyri. **(B)** Superior frontal (green) and precentral (red) sulci.

After performing a frontal craniotomy and opening the dura, we expose the frontal cortex. The frontal lobe has two horizontalized sulci and one verticalized sulcus: the superior frontal sulcus, inferior frontal sulcus, and precentral sulcus, respectively (Figure 10).

In order to correctly locate our surgical entry point, we need to recognize the exposed structures. We use the concept that the superior frontal sulcus is always the first horizontalized sulcus lateral to the interhemispheric fissure of the brain. This sulcus is, 100% of the time, interrupted by a verticalized sulcus, the precentral sulcus. Another tool that can help us locate structures in microsurgery is the path of the veins. In the frontal lobe, the veins ascend vertically and form an angle close to 90 degrees, perpendicular to the superior sagittal sinus. In contrast, in the parietal lobe, they ascend in a curvilinear trajectory and drain at an oblique angle to the superior sagittal sinus.

Our entry point is at the junction of the superior frontal sulcus, and the precentral sulcus, and the adjacent structures are the superior frontal gyrus, the middle frontal gyrus, and the precentral gyrus. Check the structures with a double confirmation using the neuro-navigator.

### 4.2 White fibers

**Figure 11:**
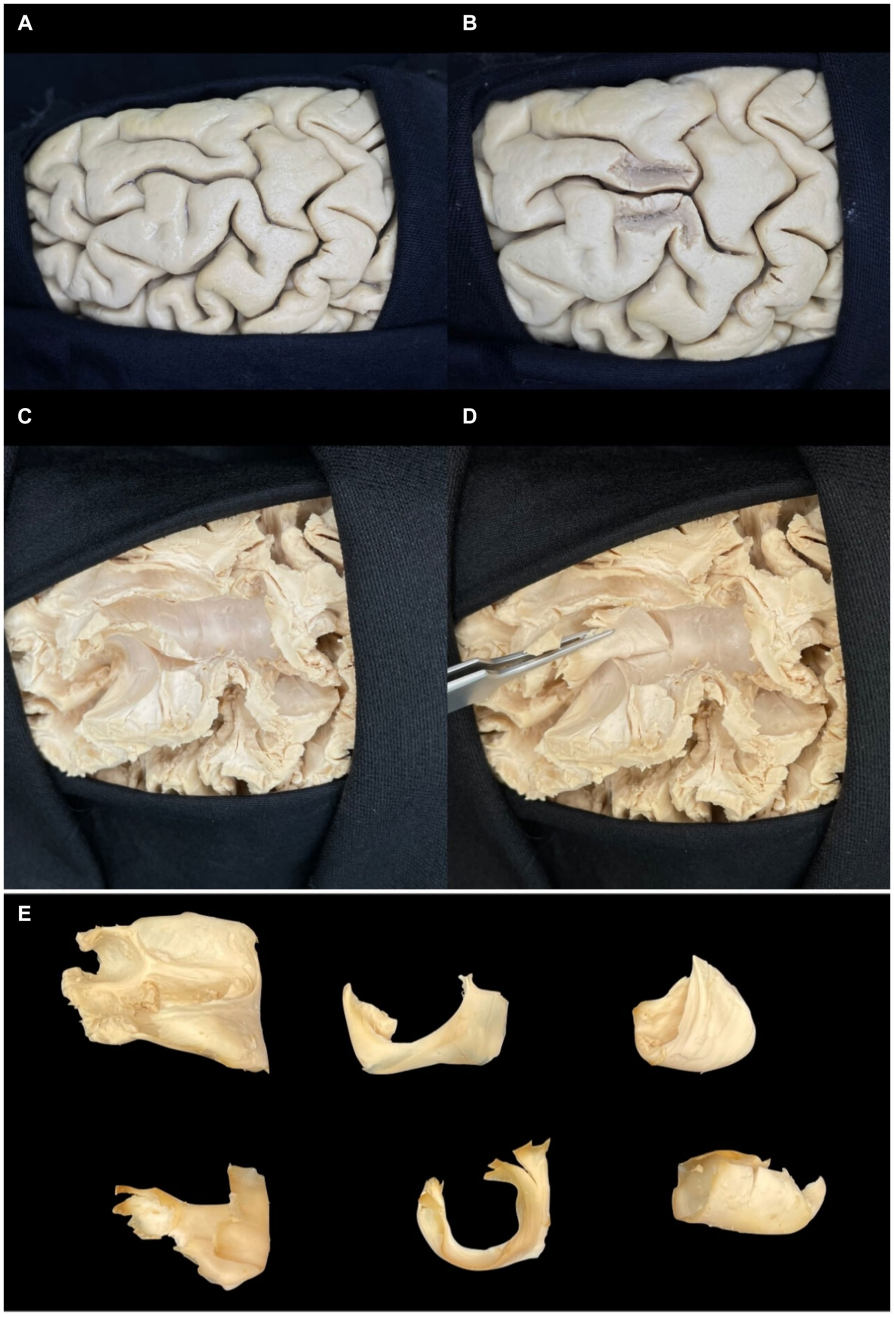
Step-by-step dissection of U-fibers. **(A)** Superior view of the superior surface of the frontal lobe. **(B)** Parasulcal minimal partial dissection of the superior and middle frontal gyrus. **(C,D)** U-fibers are forming part of the superior frontal sulcus. **(E)** White U-shaped fiber in various perspectives, showing its shape and configuration. Note the center as the deepest point to the sulcus and the ends to the gyri.

After removing the cerebral cortex, the first layer of white matter consists of short association fibers between two gyri called U-fibers (Figure 11). Then, from superficial to deep, we find the long association fibers, which connect different cerebral lobes and gather in fascicles. In the supero-inferior direction, we have the projection fibers, which connect the cortex to the brainstem through tracts, the main set of motor fibers of the internal capsule being the cortico-spinal tract.

The identification and study of white matter can be carried out by previously freezing anatomical specimens, followed by blunt dissection, a procedure known as Klingler’s Technique. Even with the gain of the surgical microscope, it is impossible to define fascicles or tracts during the surgical approach precisely. The tools to overcome this technical limitation are an exhaustive study in the neuroanatomy and microsurgery laboratory to gain a deep understanding of neuroanatomy applied to surgery and the use of magnetic resonance imaging with tractography to understand the displacement of the white fibers and the individualities of each patient. A third option is to perform surgery when the patient is awake. However, this tool is more applicable to infiltrative lesions of the brain parenchyma, which is not the case with encephalic cavernous malformations.

**Figure 12:**
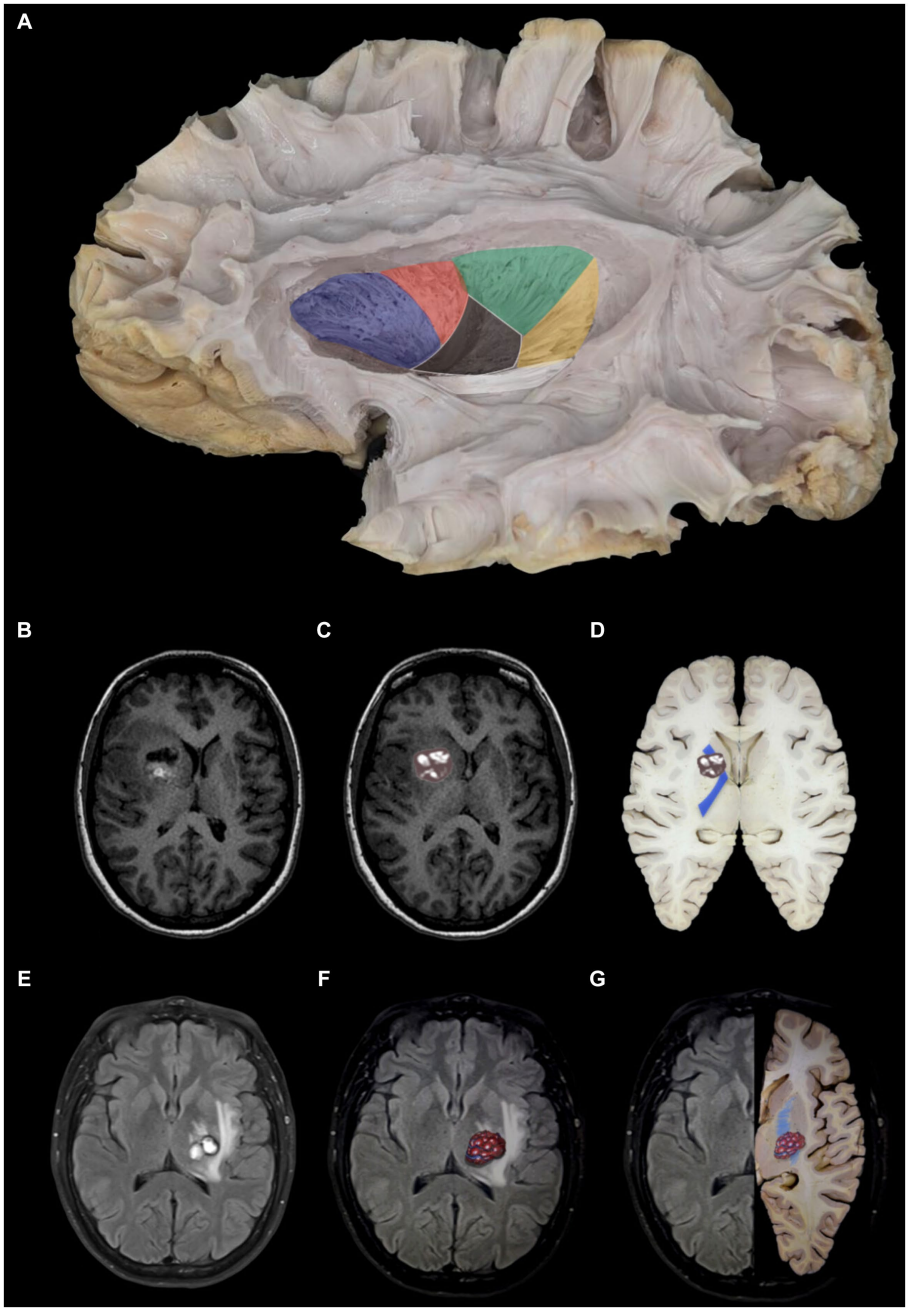
Internal capsule and relationships with cavernomas. **(A)** Anatomical representation of the internal capsule in preparation. Dissection of white fibers in left lateral view. Segmentation of the internal capsule: anterior limb (blue), knee (red), posterior limb (green), retrolenticular (yellow), and sublenticular (white). In addition to its relation to the partially resected lentiform nucleus. **(B–D)** Case of cavernoma in the anterior limb of the internal capsule. MRI—axial T1-slice. Digital overlay of right cavernoma in the region of the nuclei of the base. Anatomical piece with the representation of cavernoma and its location in the internal capsule (blue), in this case, in its anterior limb. Note its relationships lateral to the caudate head, medial to the lentiform nucleus, and posterior to the anterior horn of the lateral ventricle. The posterior limit of reference is to the knee of the internal capsule and projection to the foramen of Monro. **(E–G)** Cavernoma in the posterior limb of the internal capsule. MRI—axial T2 view. Axial MRI T2 with illustration of the brain cavernoma on the left **(C)** MRI (left) and axial section of the brain (right), illustration of the cavernoma overlying the internal capsule (blue), more precisely in its posterior limb. In addition, in the third image, we can better understand that it is in the posterior limb because it is posterior to the foramen Monro that represents the internal capsule, medial and posterior to the lentiform, lateral and anterior to the thalamus, anterior to the ventricular atrium.

The internal capsule is a more compact caudal continuation of the corona radiata, whose fibers unite to form the cerebral peduncle. It is called “internal” because it is medial to the lentiform nucleus and lies lateral to the caudate nucleus and thalamus. This structure has five portions, from anterior to posterior, which we call the anterior arm, knee, posterior arm, retrolenticular portion, and sublenticular portion (Figure 12A).

The anterior limb is lateral to the caudate nucleus and medial to the lentiform nucleus. At the same time, the posterior limb is also medial to the lentiform nucleus but medially borders the thalamus. The knee is at the junction of both arms, forming the lateral wall of the foramen Monro in the lateral ventricle, precisely at the medial apex of the globus pallidus.

Anterior limb: fibers connecting the anterior thalamus to the medial thalamus and the pontine nuclei to the frontal lobe.

Knee: cortico-thalamic and thalamocortical fibers and cortico-bulbar fibers that go to the motor nuclei of the cranial nerves.

Posterior limb: fibers connecting the thalamus with the cortex and corticospinal fibers to the motor nuclei of the trunk and upper and lower extremities.

The precentral gyrus is located superficially to the posterior leg.

Retrolenticular: fibers that curve around the posterior edge of the lentiform nucleus.

Sublenticular: fibers that pass below the lentiform nucleus. They contain fibers that make up auditory radiation (from the medial geniculate body to the primary auditory area in the transverse temporal gyrus and adjacent areas of the superior temporal gyrus) and optic radiation (from the lateral geniculate body to the walls of the calcarine sulcus).

### 4.3 The rationale behind the decision

The purpose of this article is not to discuss the best form of treatment between the conservative treatment options, radiosurgery or microsurgery while citing some arguments in favor of surgical excision, one of them is the sudden worsening of the neurological deficit, significant mass effect due to bleeding and edema, risk of cerebral herniation, risk of cranial hypertension, absence of latency between treatment and expected benefit, as with the radiotherapy option. We, therefore, opted for microsurgical treatment and will describe the rationale we used to develop our surgical approach via the superior frontal sulcus (Table 1).

**Table 1:** Comparative aspects between approaches for microsurgical treatment of lesions in the internal capsule.

Approach	Advantages	Disadvantages	Anatomical aspects
Transsylvian transinsular	Direct access route to lesions of the internal capsule	Limited access to posterior lesions, due to the presence of noble neural structures High probability of associated neurological deficit	Microsurgical dissection by these routes requires manipulation of noble neural structures, larger caliber vessels and perforators
Transcortical	Direct access route for lesions of the internal capsule	High probability of neurological deficit	
Transcallosal*	Access road with versatility, adequate working space and microsurgical vision	Limitation to access lesions with lateral extension	The positioning of the turned head, plus the gravity effect allows for better visualization
Transulcal frontal superior	Access with excellent reach in the anteroposterior axis of the internal capsule Little interposition of neurovascular structures	Limited access to sublenticular lesions	Exposure of the interhemispheric fissure provides landmarks on the transulcal dissection axis The dissection axis is oriented parallel to the fibers of the internal capsule

(*) Does not include approaches described for lesions of the anteroinferior region. *Ipsi and contralateral transcallosal approach according to lesion laterality and hemisphere dominance. These characteristics may vary comparatively according to the arrangement and displacement of the fibers in the latero-medial axis of the internal capsule.

In our method, the first step in defining the access route is to compare the MRI image with the anatomical study in the laboratory to survey the lesion correctly, recognize the structures with which it borders, and then individually define the best approach, as we did for the first case (Figures 12B–D) and second case (Figures 12E–G).

**Figure 13:**
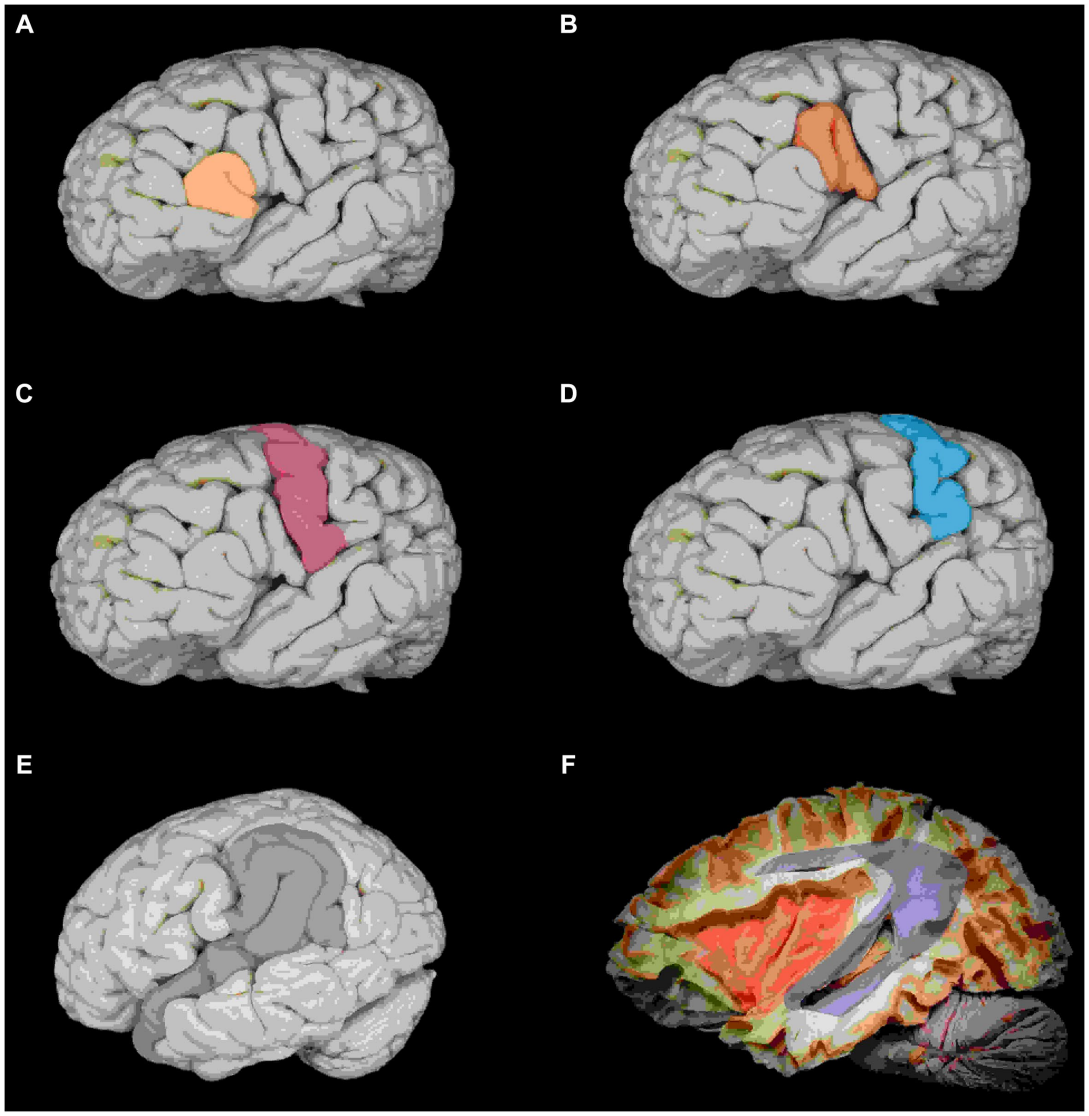
Anatomical repairs on the lateral cerebral surface to the central core. **(A)** Pars orbitalis and triangulareis (yellow). **(B)** Triangularis and opercular pars, Broca’s area (red). **(C)** Primary motor cortex (pink). **(D)** Primary sensory cortex (blue). **(E)** Identify the opercular portion of the superior temporal gyrus and supramarginal gyrus, Wernick’s area (gray). **(F)** Lateral view: arcuate fasciculus (purple) and communicating area between the Broca and Wernicke area and insula (orange).

The second step is to recognize the structures that are considered eloquent (Figure 13) and should be avoided at the risk of permanent deficit, increasing the morbidity of the procedure, especially in lesions of the dominant hemisphere, which can make the case even more complex.

**Figure 14:**
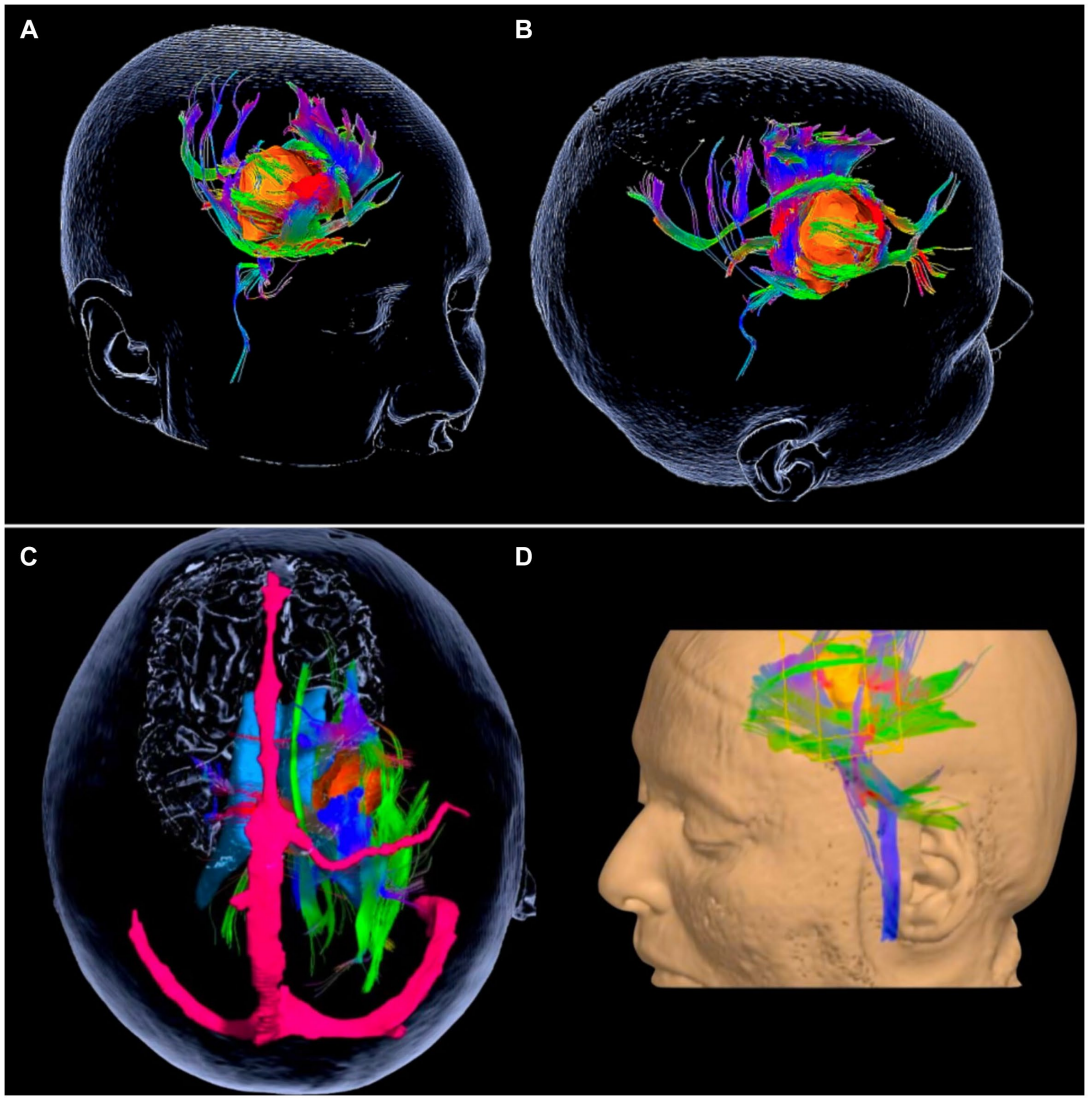
The usefulness of tractography in cavernomas. **(A,B)** Cavernoma of the anterior limb of the internal capsule. 3D reconstruction of the MRI tractography showing the relationship of the cavernosum with the nuclei of the base and adjacent white encephalic fibers. Cavernoma in orange, association fibers (green) displaced medially and laterally, projection fibers (blue and violet) displaced predominantly posteriorly and anteriorly. **(C,D)** Cavernosa of the posterior limb of the internal capsule. Cavernoma (orange). Projection fibers (blue), long association fibers, or fascicles (green). Superior sagittal sinus and vein of Trolard (in pink). **(B)** Reconstruction showing the lesion in the left frontal lobe. Note the displacement of the internal capsule projection fibers predominantly anteriorly and in smaller volumes posteriorly.

The third step is the use of 3D reconstruction tractography (Figure 14). This type of resource has an excellent indication in cavernomas since it is a lesion that displaces the fibers and does not infiltrate them, as in gliomas. Since the white fibers are not identifiable on intraoperative surgical microscopy, preoperative awareness of which direction the main tracts and fascicles are displaced can guide us in deciding on the approach.

**Figure 15:**
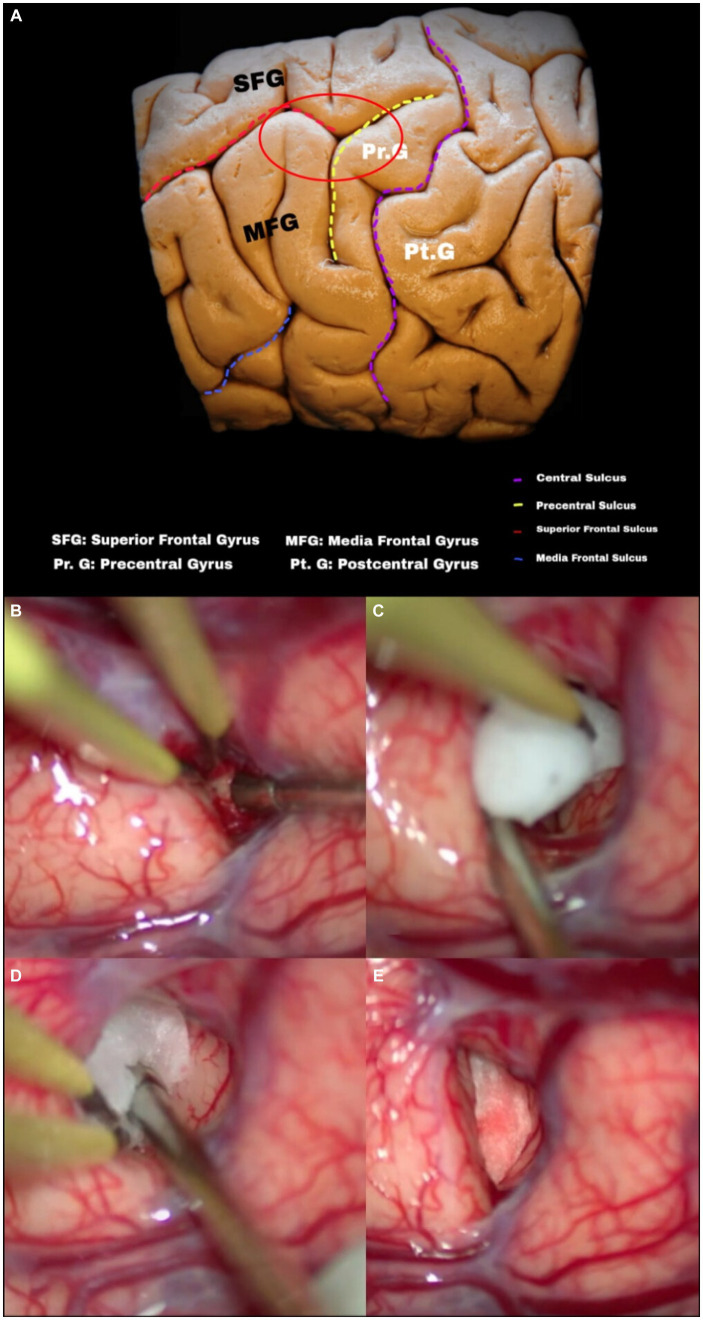
**(A)** Configuration of cerebral sulci and gyri: note the arrangement of the middle and superior frontal gyrus in the frontal lobe, as well as the superior frontal sulcus to the precentral gyrus and sulcus. Entry point and intraductal dissection. **(B–E)** Evolution of intraductal dissection (superior frontal sulcus), note the usefulness of cotton as a tool to maintain and gain space during microsurgical dissection.

The fourth step is performing the access. We used sharp dissection at the meeting point between the superior frontal sulcus and the precentral sulcus (Figure 15A). The great merit of this surgical approach is that the cortical structures are easily recognizable landmarks. To locate the superior frontal sulcus, find the first horizontalized sulcus lateral to the interhemispheric fissure. After locating the superior frontal sulcus, one should proceed posteriorly until encountering an oblique or vertically oriented sulcus, identified as the precentral sulcus.

We used intersulcal dissection to reduce cortical damage to the superior and middle frontal gyri, positioning a small piece of rolled-up absorbent cotton inside the superior frontal sulcus (Figures 15B–D) to create a real cavity where there had previously been a virtual space (Figure 15E); subarachnoid sharp dissection in experienced hands can reduce bleeding compared to traction or cortical aspiration.

Surgeons recommend intraoperative neuronavigation for two reasons: firstly, to ensure that the angle of the dissection is in the direction of the cavernoma, avoiding as much as possible inadvertent injury to displaced fibers since this is an eloquent area. Secondly, despite being a vascular surgery, we use oncological neurosurgery techniques. That is to say, we do not use the brain’s natural pathways. There is no opening of cisterns and, consequently, no major CSF drainage, reducing the intra-operative shift of the brain and ensuring more accurate navigation.

If available, ultrasound can be an auxiliary resource to increase the accuracy of lesion localization. We use the tripod of anatomy, neuronavigation, and ultrasound in 100% of our cases of deep cavernomas.

We recognize that given the unavailability of intraoperative neuronavigation in some centers, the exhaustive analysis of preparatory studies for the identification and disposition of subcortical and deep structures, the planned exposure of anatomical landmarks of the brain surface (sulci and gyri), cranial repairs, adaptation of the technique in transulcal dissection plus intraoperative ultrasound with real-time maneuverability; is an important tool to access this type of lesions in depth safely.

## 5 Conclusion

Although this work presents a small number of cases, the frontal approach via the superior frontal sulcus seems to be safe and effective for treating cavernomas of the internal capsule. The study of neuroanatomy in a microsurgery laboratory, combined with the detailed interpretation of preoperative imaging tests, is essential for developing and improving surgical approaches, especially in the case of lesions in eloquent areas.

As a positive point, we would like to highlight the reproducibility of this approach since it uses as anatomical landmarks the intersection of just two sulci, which are easily recognizable intraoperatively, ensuring wide dissemination of the technique, reaching a more significant number of neurosurgeons and patients with these challenging lesions.

On the negative side, the use of technologies that are sometimes limited in developing countries, such as tractography, intraoperative neuromonitoring, neuro-navigation, and subcortical mapping, which, although not mandatory for access, can positively impact clinical outcomes. We suggest using intraoperative ultrasound to locate the cavernoma in real-time, with good results and in a more accessible way.

## Data availability statement

The original contributions presented in the study are included in the article/supplementary material, further inquiries can be directed to the corresponding author.

## Ethics statement

The studies involving humans were approved by the Ethics and Research Committee of the Federal University of São Paulo. The studies were conducted in accordance with the local legislation and institutional requirements. The participants provided their written informed consent to participate in this study.

## Author contributions

FSP: Conceptualization, Writing – original draft, Writing – review & editing. LACJ: Investigation, Writing – review & editing. EDTA: Data curation, Writing – review & editing. RAA-T: Methodology, Writing – review & editing. LGB-S: Conceptualization, Data curation, Writing – review & editing. AFVP: Data curation, Methodology, Writing – review & editing. PHTS: Data curation, Investigation, Writing – review & editing. RW-S: Writing – original draft, Writing – review & editing. FC-N: Project administration, Supervision, Validation, Writing – review & editing.
